# Pulmonary Artery Versus Aortic Root Venting and Early Postoperative Respiratory Recovery After On-Pump Coronary Artery Bypass Grafting: A Propensity Score Overlap-Weighted Analysis

**DOI:** 10.3390/jcm15176655

**Published:** 2026-08-28

**Authors:** Seval Kılbasanlı, Esra Ertürk Tekin

**Affiliations:** 1 Department of Anesthesiology and Reanimation, Faculty of Medicine, Niğde Ömer Halisdemir University, Niğde 51240, Türkiye; 2 Department of Cardiovascular Surgery, Faculty of Medicine, Niğde Ömer Halisdemir University, Niğde 51240, Türkiye; dresraer@yahoo.com

**Keywords:** coronary artery bypass grafting, cardiac surgery, cardiopulmonary bypass, pulmonary artery venting, aortic root venting, early extubation, fast-track recovery, propensity-score overlap weighting

## Abstract

**Background/Objectives:** Direct comparative evidence regarding aortic root and pulmonary artery venting during on-pump coronary artery bypass grafting (CABG) remains limited. We evaluated whether pulmonary artery venting was associated with improved early postoperative respiratory recovery compared with aortic root venting. **Methods:** This single-center retrospective cohort included 488 adults undergoing on-pump CABG between January 2020 and January 2025 (aortic root vent, *n* = 316; pulmonary artery vent, *n* = 172). The primary outcome was delayed extubation beyond 8 h. Propensity-score overlap weighting based on 22 covariates addressed baseline confounding. Confidence intervals and *p* values were obtained from 5000 stratified nonparametric bootstrap samples with refitting of the propensity-score model and recalculation of overlap weights in each sample. **Results:** Extubation-time data were available for 486 patients. As expected with overlap weighting based on a logistic propensity-score model, exact mean balance was achieved for the covariates included in the model (absolute standardized mean differences < 0.001). The overlap-weighted risk of delayed extubation was 51.4% in the aortic root vent group and 41.1% in the pulmonary artery vent group; the adjusted association was not statistically significant (odds ratio [OR] 0.66, 95% confidence interval [CI] 0.37–1.10; *p* = 0.110). Pulmonary artery venting was associated with lower odds of fast-track failure (OR 0.53, 95% CI 0.30–0.89), the respiratory composite outcome (OR 0.35, 95% CI 0.17–0.64), and postoperative inotrope requirement (OR 0.39, 95% CI 0.14–0.87), together with a higher arterial oxygen partial pressure (PaO2) at extubation (mean difference 7.08 mmHg, 95% CI 0.72–12.89). **Conclusions:** The primary outcome of delayed extubation did not differ significantly between venting strategies after overlap weighting. Favorable associations across several secondary outcomes suggest a possible improvement in broader early postoperative recovery with pulmonary artery venting; these exploratory findings require prospective confirmation.

## 1. Introduction

Early postoperative respiratory recovery is a central component of fast-track pathways after coronary artery bypass grafting (CABG). Prolonged mechanical ventilation may delay mobilization and intensive care unit discharge, whereas early extubation can be performed safely in appropriately selected patients without an apparent increase in major postoperative complications [1]. Fast-track cardiac care protocols have been associated with shorter times to extubation and reduced intensive care unit length of stay while maintaining clinical safety [2]. The contemporary enhanced-recovery literature and recommendations emphasize a multidisciplinary perioperative approach to facilitate timely recovery after adult cardiac surgery [3,4], and implementation of a structured enhanced-recovery program has been associated with shorter intensive care unit and hospital stays [5]. Perioperative factors that may influence pulmonary function, oxygenation, and readiness for extubation therefore remain clinically relevant in patients undergoing on-pump CABG.

Cardiopulmonary bypass (CPB) may impair postoperative pulmonary function through reduced pulmonary blood flow, ischemia–reperfusion injury, systemic inflammatory activation, increased pulmonary vascular permeability, atelectasis, and altered gas exchange [6,7,8,9]. During cardiopulmonary bypass, blood returning to the left side of the heart through the bronchial circulation and other collateral pathways may cause progressive cardiac distension and interfere with myocardial protection. Venting is therefore used to decompress the left heart, maintain a relatively bloodless operative field, and facilitate de-airing before separation from bypass. A pulmonary artery vent can achieve indirect left-heart decompression by removing blood from the pulmonary circulation and has been shown to reduce left-sided cardiac pressures effectively during coronary artery bypass surgery [10].

Direct comparisons of aortic root and pulmonary artery venting remain limited. In a retrospective study of 301 patients undergoing on-pump CABG, pulmonary artery venting was associated with higher postoperative arterial oxygen partial pressure (PaO2), lower requirements for nasotracheal aspiration and bronchodilator therapy, and fewer reintubations, although postoperative intubation duration was similar between groups [11]. A subsequent study reported a lower incidence of postoperative atrial fibrillation with pulmonary artery venting than with aortic root venting [12]. However, these reports primarily evaluated individual pulmonary or rhythm outcomes and did not specifically examine early respiratory recovery within a contemporary fast-track framework using robust adjustment for baseline differences. The present study therefore compared pulmonary artery and aortic root venting in patients undergoing on-pump CABG, with delayed extubation beyond 8 h as the primary outcome and fast-track failure, respiratory complications, postoperative atrial fibrillation, and other early clinical outcomes as secondary endpoints. We hypothesized that pulmonary artery venting would be associated with more favorable early postoperative respiratory recovery.

## 2. Materials and Methods

### 2.1. Study Design and Population

This single-center retrospective observational cohort study was conducted at Mersin City Training and Research Hospital. Adult patients who underwent isolated on-pump CABG between January 2020 and January 2025 and in whom either aortic root or pulmonary artery venting was used were evaluated. No patient underwent concomitant valve surgery or another major cardiac surgical procedure. Patients were classified according to the venting strategy documented in the operative records as the aortic root vent group or the pulmonary artery vent group.

The final cohort comprised 488 patients, including 316 patients in the aortic root vent group and 172 patients in the pulmonary artery vent group. All eligible patients with an identifiable venting strategy and sufficient perioperative clinical data were included. Missing outcome data were handled using outcome-specific available-case analysis; therefore, patients were not excluded from the overall cohort solely because an individual postoperative measurement was unavailable. Extubation-time data were available for 486 patients. Patients were followed from surgery through to hospital discharge.

### 2.2. Ethical Considerations

The study protocol was approved by the Mersin University Clinical Research Ethics Committee on 5 February 2025 (decision no. 2025/137). The study was conducted in accordance with the Declaration of Helsinki and applicable national regulations. Institutional permission was obtained from Mersin City Training and Research Hospital before data collection. Owing to the retrospective design, separate study-specific informed consent was not obtained. Only routinely collected clinical records were reviewed, and all patient data were anonymized before statistical analysis.

### 2.3. Data Collection and Study Variables

Demographic, clinical, operative, and postoperative data were retrospectively obtained from hospital records. Baseline variables included age, sex, chronic obstructive pulmonary disease, diabetes mellitus, hypertension, hyperlipidemia, previous myocardial infarction, smoking history, preoperative medication use, blood urea nitrogen (BUN), serum creatinine, hematocrit, left ventricular ejection fraction, and New York Heart Association (NYHA) functional class.

Operative variables included emergency surgery, left main coronary artery disease, use of the left internal mammary artery, number of distal anastomoses, aortic cross-clamp time, cardiopulmonary bypass time, and intraoperative fluid balance. Postoperative variables included time to extubation, intensive care unit and hospital length of stay, drainage volume, transfusion requirement, postoperative atrial fibrillation, inotrope requirement, low cardiac output, acidosis, diaphragmatic paralysis, nasotracheal aspiration, inhaled bronchodilator requirement, reintubation, stroke, and in-hospital mortality. Arterial oxygen partial pressure and arterial oxygen saturation (SaO2) measured at or immediately before extubation were also recorded.

All variables were defined according to the information documented in the clinical records. Detailed definitions and coding of the study variables are provided in Table S1.

### 2.4. Venting Strategies and Perioperative Management

All patients underwent on-pump CABG using institutional surgical and cardiopulmonary bypass protocols. Patients were categorized according to the venting technique documented in the operative record. During the study period, CABG procedures were performed by two surgical teams, each using a distinct established venting strategy. Accordingly, venting strategy primarily reflected the routine operative practice of the surgical team rather than patient-specific intraoperative selection.

Standard intermittent antegrade cold blood cardioplegia was used in both groups, with repeat doses administered at approximately 20 min intervals, together with topical cooling. Moderate systemic hypothermia (28–32 °C) was maintained during CPB. In the aortic root vent group, cardiac decompression was achieved using an aortic root vent, and antegrade cardioplegia was delivered through the aortic root cannula. In the pulmonary artery vent group, a vent catheter placed in the main pulmonary artery was used for decompression, while antegrade cardioplegia was administered through a separate needle placed in the ascending aorta.

In the pulmonary artery vent strategy, proximal anastomoses were performed during a single period of aortic cross-clamping. In contrast, in the aortic root vent strategy, the aortic cross-clamp was removed before proximal anastomoses, which were subsequently performed using a side-biting aortic clamp. De-airing was performed by direct needle aspiration through the left ventricular apex. No additional left ventricular vent through the right superior pulmonary vein was routinely used, and carbon dioxide field flooding was not employed.

During CPB, the lungs were left deflated, followed by lung recruitment after separation from CPB. No pulmonary artery vent-site complications were documented, and no reoperation for bleeding was attributable to the pulmonary artery vent site. Perioperative anesthetic management, postoperative mechanical ventilation, and intensive care unit care were performed according to routine institutional protocols applicable during the study period. Decisions regarding ventilator weaning and tracheal extubation were made by the responsible clinical team based on hemodynamic stability, adequate oxygenation and ventilation, recovery of consciousness, and the absence of clinically significant bleeding or other conditions requiring continued mechanical ventilation.

### 2.5. Study Outcomes and Definitions

The primary outcome was delayed extubation, defined as tracheal extubation occurring more than 8 h after completion of surgery. Time to extubation was calculated as the interval between completion of surgery, immediately before transfer to the intensive care unit, and successful removal of the endotracheal tube. Patients with missing extubation-time data were excluded only from analyses involving this outcome.

The principal secondary outcome was fast-track failure, defined as the occurrence of at least one of the following: delayed extubation beyond 8 h, intensive care unit stay longer than 1 day, or postoperative reintubation. A respiratory composite outcome was defined as the presence of at least one of the following: diaphragmatic paralysis, requirement for nasotracheal aspiration, requirement for inhaled bronchodilator treatment, or reintubation.

Other secondary outcomes included continuous extubation time, intensive care unit and hospital length of stay, postoperative atrial fibrillation, inotrope requirement, low cardiac output, acidosis, revision surgery, postoperative transfusion requirement, drainage volume, PaO_2_ and SaO_2_ at extubation, stroke, reintubation, and in-hospital mortality. Postoperative atrial fibrillation was defined as newly documented atrial fibrillation after surgery. Reintubation was defined as the requirement for repeat tracheal intubation after initial successful extubation.

To evaluate the robustness of the primary analysis, delayed extubation was additionally examined using alternative thresholds of more than 6 h and more than 12 h. Detailed definitions and coding of all study variables are presented in Table S1.

### 2.6. Statistical Analysis

Continuous variables were summarized as mean ± standard deviation or median [interquartile range], according to their distribution, and categorical variables were presented as numbers and percentages. In unadjusted analyses, continuous variables were compared using the Mann–Whitney U test, whereas categorical variables were compared using Pearson’s chi-square test or Fisher’s exact test, as appropriate. Baseline differences between venting groups were primarily assessed using absolute standardized mean differences (SMDs), with an absolute SMD below 0.10 considered indicative of adequate covariate balance.

Because venting strategy was not randomly assigned, propensity-score overlap weighting was used to reduce measured baseline confounding [13,14,15,16]. The propensity score represented the probability of receiving pulmonary artery rather than aortic root venting and was estimated using multivariable logistic regression. The model included age, sex, chronic obstructive pulmonary disease, diabetes mellitus, hypertension, hyperlipidemia, previous myocardial infarction, smoking history, beta-blocker use, calcium-channel blocker use, angiotensin-converting enzyme inhibitor use, aspirin use, clopidogrel use, preoperative blood urea nitrogen, creatinine, hematocrit, left ventricular ejection fraction, New York Heart Association (NYHA) functional class, number of distal anastomoses, emergency surgery, left main coronary artery disease, and left internal mammary artery use. Although NYHA functional class showed limited variability in this cohort, it was retained in the propensity-score model because it represents a clinically relevant preoperative measure of functional status and was available for all patients. Emergency surgical status was determined preoperatively by the surgical team and was therefore treated as a pre-exposure covariate. The number of distal anastomoses and left internal mammary artery use represented the procedures actually performed and recorded intraoperatively and were included in the original propensity-score model to account for measured differences in operative complexity. Because these variables are finalized during the operation and their temporal relationship to the venting strategy is therefore less clear than that of preoperative covariates, a sensitivity analysis was performed using an alternative propensity-score model excluding both variables. Aortic cross-clamp time, cardiopulmonary bypass time, and intraoperative fluid balance were not included in either model because they occur downstream of the venting strategy and may be affected by the operative course. Continuous covariates were standardized before model fitting.

For overlap weighting, patients in the aortic root vent group were assigned a weight equal to their propensity score, whereas patients in the pulmonary artery vent group were assigned a weight equal to one minus their propensity score. This approach emphasizes patients with the greatest clinical overlap between treatment groups and downweighs observations with extreme propensity scores [13,14,15]. The corresponding estimand was the average treatment effect in the overlap population (ATO), which emphasizes patients with measured baseline characteristics shared across the two observed strategy groups rather than patients at the extremes of the treatment-assignment distribution. Covariate balance was reassessed after weighting using absolute SMDs. Because overlap weighting based on a logistic propensity-score model achieves exact balance in the first moments of covariates included in the model, additional balance diagnostics were performed for squared terms of the continuous covariates and for a selected set of clinically relevant interactions (age × left ventricular ejection fraction, age × emergency surgery, diabetes mellitus × hypertension, chronic obstructive pulmonary disease × smoking, and left ventricular ejection fraction × emergency surgery). Effective sample sizes were calculated using the Kish method. No patients were excluded or trimmed on the basis of the propensity score. To further characterize the extent of empirical overlap, propensity-score ranges were reported separately for each venting group, and near-extreme propensity scores were descriptively defined as values <0.05 or >0.95.

For binary outcomes, overlap-weighted risks were estimated for each group, and treatment effects were expressed as risk differences, risk ratios, and odds ratios comparing pulmonary artery with aortic root venting. The principal effect measure reported for binary outcomes was the adjusted odds ratio. For continuous outcomes, overlap-weighted means and adjusted mean differences were calculated. Ninety-five percent confidence intervals were obtained using the percentile method from 5000 stratified nonparametric bootstrap samples. Patients were resampled with replacement separately within each venting group, and the propensity-score model was refitted, and overlap weights were recalculated in every bootstrap sample. Two-sided bootstrap *p* values were calculated as twice the smaller of the proportions of bootstrap estimates on either side of the null value; ratio measures were evaluated on the logarithmic scale. A finite-sample correction of one was applied to the numerator and denominator of these proportions. A fixed random seed (20260817) was used to ensure reproducibility.

Missing data were handled using outcome-specific available-case analysis. Because all covariates included in the propensity-score model were complete, multiple imputations were not performed. The valve-disease variable was excluded from statistical modeling because 87.3% of observations were missing. As an additional sensitivity analysis addressing the temporal ordering of operative covariates, the primary analysis was repeated using an alternative propensity-score model that excluded the number of distal anastomoses and left internal mammary artery use while retaining all other covariates. Sensitivity analyses repeated the primary weighted analysis using delayed-extubation thresholds of more than 6 h and more than 12 h. All eligible patients were included; therefore, no prospective sample-size calculation was performed. Statistical tests were two-sided, and *p* < 0.05 was considered statistically significant. No adjustment for multiple comparisons was applied; analyses of secondary outcomes were considered exploratory. Statistical analyses were performed in Python version 3.13.5 (Python Software Foundation, Wilmington, DE, USA) using the open-source packages NumPy version 2.3.5, SciPy version 1.17.0, pandas version 2.2.3, and statsmodels version 0.14.6; the analysis code is provided as Supplementary File S1. The extent and handling of missing data are summarized in Table S2.

## 3. Results

### 3.1. Study Population and Baseline Characteristics

A total of 488 patients undergoing on-pump CABG were included. Of these, 316 patients received aortic root venting and 172 received pulmonary artery venting. Extubation-time data were available for 486 patients, including 314 patients in the aortic root vent group and all 172 patients in the pulmonary artery vent group (Figure S1).

Both surgical teams were active concurrently throughout the study period, and both venting strategies were represented in every calendar year. The annual distribution of aortic root versus pulmonary artery venting was 38 versus 22 cases in 2020, 43 versus 27 in 2021, 68 versus 37 in 2022, 74 versus 41 in 2023, 84 versus 41 in 2024, and 9 versus 4 in January 2025, respectively. There was no evidence of a significant change in the relative distribution of venting strategies across calendar years (χ^2^ = 0.84, *p* = 0.974), indicating that the two strategies did not represent sequential eras of practice (Table S5).

Before propensity-score weighting, several clinically relevant differences were observed between groups. Patients in the pulmonary artery vent group were less frequently male and had higher prevalences of diabetes mellitus, hyperlipidemia, and previous myocardial infarction, whereas hypertension, calcium-channel blocker use, angiotensin-converting enzyme inhibitor use, and clopidogrel use were more frequent in the aortic root vent group.

**Figure 1:**
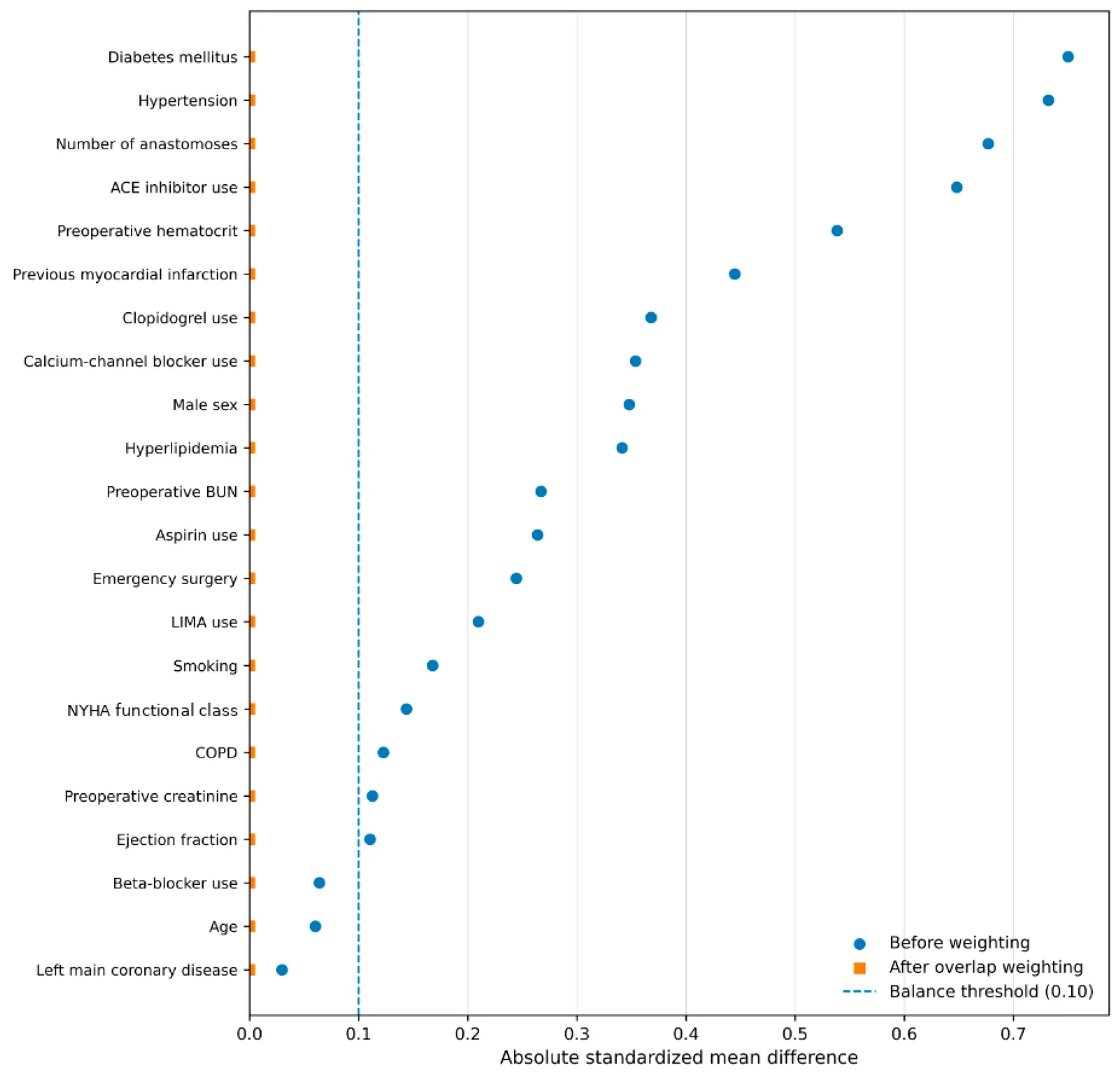
Covariate balance before and after propensity-score overlap weighting. Absolute standardized mean differences are shown for the 22 covariates included in the propensity-score model. The dashed vertical line indicates the predefined balance threshold of 0.10. Abbreviations: ACE, angiotensin-converting enzyme; BUN, blood urea nitrogen; COPD, chronic obstructive pulmonary disease; LIMA, left internal mammary artery; NYHA, New York Heart Association.

The pulmonary artery vent group had a higher preoperative hematocrit level, whereas the number of distal anastomoses was greater in the aortic root vent group. The wide standard deviation of preoperative BUN in the aortic root vent group reflected a right-skewed distribution with high outlying values rather than a calculation error; the unadjusted median [IQR] BUN was 19 [15–28] mg/dL in the aortic root vent group and 18 [15–24] mg/dL in the pulmonary artery vent group. The largest pre-weighting imbalances were observed for diabetes mellitus, hypertension, number of distal anastomoses, angiotensin-converting enzyme inhibitor use, and preoperative hematocrit. As expected from the properties of overlap weighting based on a logistic propensity-score model, exact balance in the means of the covariates included in the propensity model was achieved after weighting, with absolute SMDs below 0.001 (Table 1 and Figure 1). This exact balance pertains to the first moments of the modeled covariates and should not be interpreted as evidence of balance in higher-order terms, interactions, or unmeasured characteristics.

Additional balance diagnostics demonstrated that exact first-moment balance did not extend uniformly to higher-order covariate structure. Across the examined squared terms and clinically relevant interactions, the maximum absolute SMD was 0.331. The largest residual imbalances were observed for the squared terms of number of distal anastomoses (SMD = 0.331), preoperative hematocrit (SMD = 0.305), and preoperative creatinine (SMD = 0.302), and for the diabetes mellitus × hypertension interaction (SMD = 0.258). These findings reinforce that the exact mean balance after overlap weighting should not be interpreted as complete balance of the underlying covariate distributions. Conventional propensity-score model diagnostics and propensity-score distributions are presented in Table S3 and Figure S2, respectively. Propensity scores ranged from 0.000007 to 0.990 in the aortic root vent group and from 0.005600 to 0.997 in the pulmonary artery vent group. Near-extreme propensity scores (<0.05 or >0.95) were observed in 108/316 (34.2%) and 35/172 (20.3%) patients, respectively (Table S3; Figure S2).

The number of distal anastomoses and left internal mammary artery use represented the procedures actually performed and recorded intraoperatively and were included in the original propensity-score model to account for measured differences in operative complexity. Because these variables are finalized during the operation and their temporal relationship to the venting strategy is therefore less clear than that of preoperative covariates, a sensitivity analysis was performed using an alternative propensity-score model excluding both variables. Aortic cross-clamp time, cardiopulmonary bypass time, and intraoperative fluid balance were not included in either model because they occur downstream of the venting strategy and may be affected by the operative course.

### 3.2. Intraoperative Characteristics and Unadjusted Postoperative Outcomes

In unadjusted analyses, aortic cross-clamp time was longer in the pulmonary artery vent group than in the aortic root vent group (60 [45–71.5] vs. 44 [35–61.2] min; *p* < 0.001), whereas cardiopulmonary bypass time was slightly shorter (85 [69.5–99] vs. 90 [72–114] min; *p* = 0.019). Patients in the pulmonary artery vent group received fewer postoperative transfusion units (1 [0–2] vs. 2 [1–2]; *p* < 0.001), although their intraoperative fluid balance was higher (525 [150–800] vs. 100 [−300 to 662.5] mL; *p* < 0.001).

Time to extubation was shorter in the pulmonary artery vent group (7.5 [6–10] vs. 9 [6–11] h; *p* = 0.001). The unadjusted incidence of delayed extubation was 37.2% in the pulmonary artery vent group and 53.8% in the aortic root vent group (*p* < 0.001), whereas fast-track failure occurred in 40.7% and 60.5% of patients, respectively (*p* < 0.001). The distribution of intensive care unit length of stay was also lower in the pulmonary artery vent group (1 [1–1] vs. 1 [1–2] days; *p* < 0.001), whereas median hospital stay was slightly longer (7 [6–7] vs. 6 [5–7.2] days; *p* = 0.004). The distribution of extubation times by venting strategy is shown in Figure S3.

Postoperative atrial fibrillation (9.9% vs. 19.6%; *p* = 0.005), the respiratory composite outcome (11.0% vs. 43.4%; *p* < 0.001), and inotrope requirement (8.1% vs. 24.1%; *p* < 0.001) were less frequent in the pulmonary artery vent group. PaO_2_ and SaO_2_ values at extubation were also higher in the pulmonary artery vent group. Among the individual components of the respiratory composite outcome, nasotracheal aspiration (3.5% vs. 30.7%) and inhaled bronchodilator requirement (9.3% vs. 37.3%) were substantially less frequent in the pulmonary artery vent group than in the aortic root vent group. Diaphragmatic paralysis was uncommon, occurring in 3/172 patients (1.7%) in the pulmonary artery vent group and in none of the 316 patients in the aortic root vent group. Reintubation occurred in 16/316 patients (5.1%) in the aortic root vent group and in none of the patients in the pulmonary artery vent group. Because individual components could co-occur within the same patient, component frequencies were not mutually exclusive. Complete unadjusted intraoperative and postoperative findings are presented in Table 2. Overlap-weighted estimates for prespecified secondary outcomes not included in Table 3, together with individual components of the respiratory and fast-track composite outcomes, are presented in Table S6. 

### 3.3. Overlap-Weighted Adjusted Outcomes

After propensity-score overlap weighting, the adjusted risk of delayed extubation (>8 h) was 51.4% in the aortic root vent group and 41.1% in the pulmonary artery vent group. Although pulmonary artery venting was associated with lower odds of delayed extubation, the difference did not reach statistical significance (adjusted odds ratio [OR] 0.66, 95% confidence interval [CI] 0.37–1.10; *p* = 0.110). Adjusted mean extubation time was 9.00 h in the aortic root vent group and 8.50 h in the pulmonary artery vent group (adjusted mean difference −0.49 h, 95% CI −1.48 to 0.41; *p* = 0.269).

The Kish effective sample sizes after overlap weighting were 135.0 for the aortic root vent group and 97.1 for the pulmonary artery vent group, compared with nominal group sizes of 316 and 172, respectively. This reduction reflects the downweighting of patients with extreme propensity scores and the focus of the overlap estimand on patients with baseline characteristics shared across the two groups. The change from the crude group difference to the adjusted estimate reflects adjustment for substantial measured confounding and the change in target population, whereas the reduced effective sample sizes decrease statistical precision. Accordingly, the confidence interval around the neutral primary estimate does not exclude a potentially clinically meaningful difference between strategies. The resulting overlap population represented patients with baseline characteristics shared across the two original groups; for example, the overlap-weighted prevalences of diabetes mellitus, hypertension, and previous myocardial infarction were 49.4%, 56.3%, and 22.4%, respectively, with a mean age of 61.5 years (Table 1). Thus, the adjusted estimates are most applicable to patients whose baseline characteristics were compatible with either group rather than to patients at the extremes of the original treatment-assignment distribution.

Fast-track failure remained significantly less frequent after weighting, with adjusted risks of 58.0% in the aortic root vent group and 42.2% in the pulmonary artery vent group (adjusted OR 0.53, 95% CI 0.30–0.89; *p* = 0.016). The pulmonary artery vent group also had lower odds of the respiratory composite outcome (15.9% vs. 35.0%; adjusted OR 0.35, 95% CI 0.17–0.64; *p* < 0.001) and postoperative inotrope requirement (6.0% vs. 13.9%; adjusted OR 0.39, 95% CI 0.14–0.87; *p* = 0.027).

**Figure 2:**
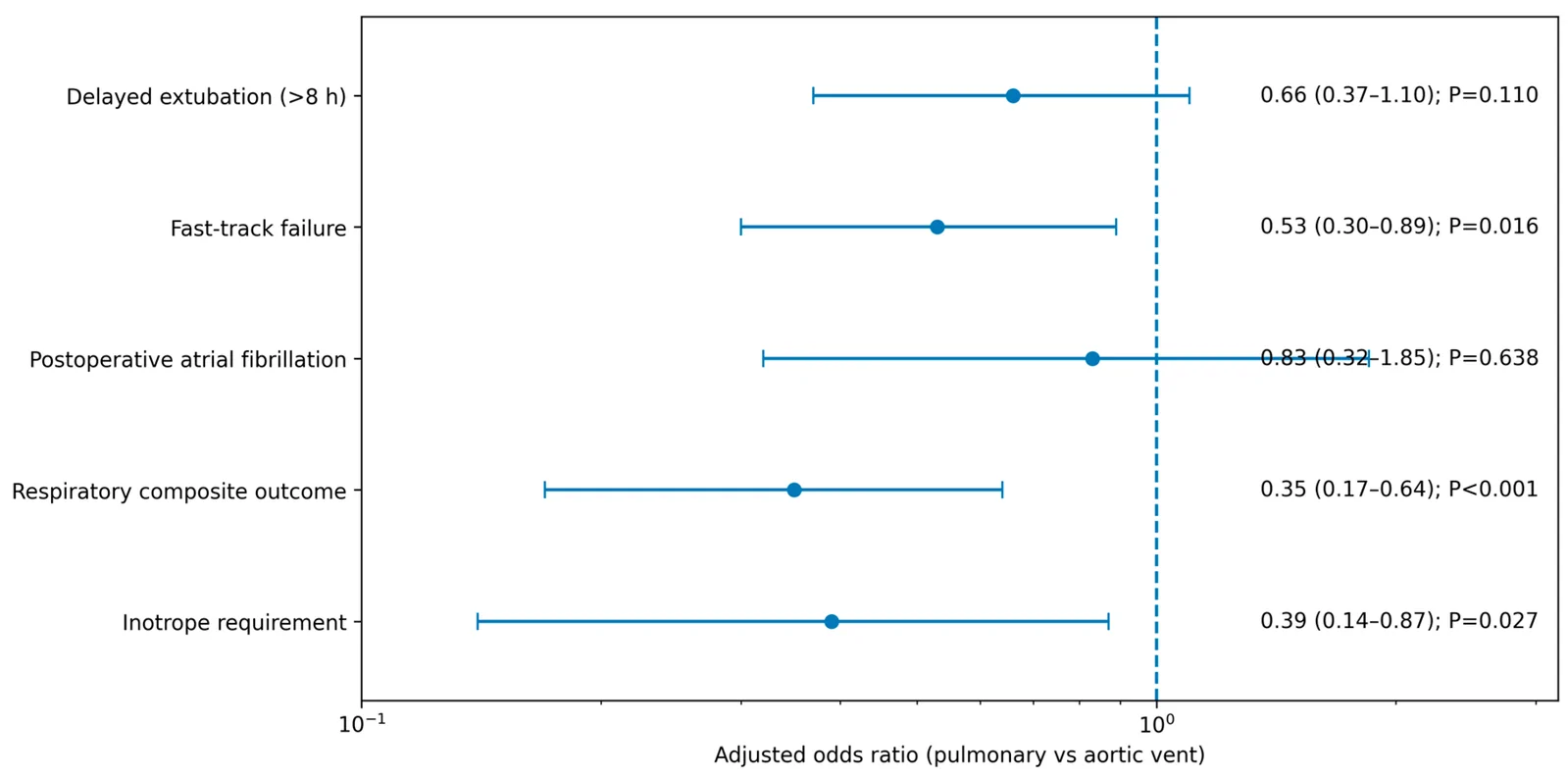
Overlap-weighted clinical outcomes. Adjusted odds ratios compare pulmonary artery venting with aortic root venting. Points indicate adjusted odds ratios, horizontal lines indicate 95% confidence intervals, and the dashed vertical line indicates the null value of 1.0.

No significant between-group difference was observed in postoperative atrial fibrillation (11.4% vs. 13.4%; adjusted OR 0.83, 95% CI 0.32–1.85; *p* = 0.638), intensive care unit length of stay (adjusted mean difference −0.19 days, 95% CI −0.39 to 0.03; *p* = 0.085), or hospital length of stay (adjusted mean difference 0.22 days, 95% CI −0.34 to 0.75; *p* = 0.440). PaO_2_ at extubation was higher in the pulmonary artery vent group (105.85 vs. 98.77 mmHg; adjusted mean difference 7.08 mmHg, 95% CI 0.72–12.89; *p* = 0.027). Adjusted outcome estimates are presented in Table 3 and Figure 2.

### 3.4. Sensitivity Analyses

The direction of association for delayed extubation was not consistent across alternative time thresholds. Using a threshold of more than 6 h, the adjusted risks were 70.5% in the aortic root vent group and 72.9% in the pulmonary artery vent group, with no significant difference between groups (adjusted OR 1.13, 95% CI 0.64–1.98; *p* = 0.667). At the primary threshold of more than 8 h, pulmonary artery venting was associated with a lower adjusted risk of delayed extubation, although the difference remained statistically non-significant (41.1% vs. 51.4%; adjusted OR 0.66, 95% CI 0.37–1.10; *p* = 0.110). Similarly, when delayed extubation was defined as more than 12 h, the adjusted risks were 8.1% in the pulmonary artery vent group and 11.6% in the aortic root vent group (adjusted OR 0.67, 95% CI 0.23–1.63; *p* = 0.339). Thus, the point estimate at the 6 h threshold was on the opposite side of unity from those at the 8- and 12 h thresholds, indicating that the direction of association was unstable at the earliest threshold. All estimates were imprecise and did not reach statistical significance (Table S4).

In the additional sensitivity analysis excluding the number of distal anastomoses and left internal mammary artery use from the propensity-score model, the overlap-weighted risks of delayed extubation > 8 h were 50.2% in the aortic root vent group and 41.9% in the pulmonary artery vent group. The association remained statistically non-significant (adjusted OR 0.71, 95% CI 0.41–1.19; *p* = 0.187), indicating that the primary conclusion was not materially affected by exclusion of these intraoperatively determined covariates.

## 4. Discussion

### 4.1. Principal Findings

In this single-center retrospective cohort of patients undergoing on-pump CABG, pulmonary artery venting was associated with a more favorable pattern of early postoperative recovery than aortic root venting after adjustment for measured baseline differences. Although the primary outcome of delayed extubation beyond 8 h was numerically less frequent with pulmonary artery venting, the adjusted association did not reach statistical significance. In contrast, pulmonary artery venting was significantly associated with lower odds of fast-track failure, the respiratory composite outcome, and postoperative inotrope requirement, as well as a modestly higher PaO_2_ at extubation. No significant adjusted differences were observed in continuous extubation time, intensive care unit or hospital length of stay, or postoperative atrial fibrillation. Taken together, these findings suggest that pulmonary artery venting may be associated with improved early respiratory and overall fast-track recovery; however, the absence of a statistically significant effect on the primary outcome warrants cautious interpretation.

The marked unadjusted differences in reintubation and in-hospital mortality also warrant particular caution. Reintubation occurred in 16 patients (5.1%) in the aortic root vent group and in none of the patients in the pulmonary artery vent group, while in-hospital mortality was 5.1% (16/316) and 0.6% (1/172), respectively. These large crude differences are unlikely to be attributable to the anatomical venting route alone and should not be interpreted as evidence of a causal effect of venting strategy. Rather, together with the substantial baseline differences between groups, they reinforce the possibility of residual unmeasured differences in case mix and team-specific perioperative practice despite adjustment for measured covariates.

### 4.2. Extubation and Fast-Track Recovery

Early tracheal extubation, commonly defined as extubation within 8 h after cardiac surgery, is an established component of fast-track cardiac care and can be implemented safely in appropriately selected patients [1,2,3,4,5]. Prolonged postoperative ventilation after CABG is associated with substantial morbidity and mortality [17]. In the present study, the marked unadjusted difference in delayed extubation was attenuated after overlap weighting. Although the adjusted odds of extubation beyond 8 h were approximately 34% lower with pulmonary artery venting, the confidence interval included the null value. This finding suggests a potentially favorable association but does not provide conclusive evidence that venting strategy independently determines extubation timing.

Conversely, fast-track failure remained significantly less frequent in the pulmonary artery vent group after adjustment. Because this composite outcome incorporated delayed extubation, intensive care unit stay longer than 1 day, and reintubation, the result may indicate an association with broader postoperative recovery rather than with extubation time alone. However, composite outcomes may be influenced disproportionately by their more frequent components, and this finding should therefore be interpreted together with individual outcome estimates. Our results extend the previous fast-track literature by suggesting that an intraoperative surgical factor, in addition to anesthetic and postoperative care protocols, may contribute to early recovery after CABG.

### 4.3. Respiratory Outcomes and Oxygenation

The most pronounced adjusted association was observed for the respiratory composite outcome, which was less frequent in the pulmonary artery vent group. This finding was accompanied by lower unadjusted rates of nasotracheal aspiration, inhaled bronchodilator requirement, and reintubation. In addition, PaO_2_ at extubation remained modestly higher after overlap weighting. Although statistically significant, the adjusted between-group difference in PaO_2_ was modest (7.08 mmHg), and its clinical significance remains uncertain. These findings are consistent with the earlier study by Haberal et al., in which pulmonary artery venting was associated with better postoperative oxygenation and reduced requirements for nasotracheal aspiration, bronchodilator therapy, and reintubation compared with aortic root venting, while intubation duration and lengths of intensive care unit and hospital stay were similar between groups [11].

A potential explanation is that pulmonary artery venting provides effective indirect decompression of the left heart while removing blood from the pulmonary circulation during cardiopulmonary bypass, which may limit pulmonary vascular engorgement and facilitate postoperative gas exchange [10]. Contemporary cardiopulmonary bypass guidance continues to recognize cardiac and great-vessel venting as an established component of intraoperative decompression and operative-field management [18]. However, the present study did not include direct measurements of pulmonary congestion, lung compliance, alveolar–arterial oxygen gradients, or PaO_2_/fraction of inspired oxygen (FiO2) ratios. Moreover, the respiratory composite was driven largely by nasotracheal aspiration and inhaled bronchodilator use, both of which were clinician-initiated supportive interventions rather than uniformly adjudicated pulmonary complications. Their use may therefore reflect differences in intensive care practice, team-specific management, or other unmeasured factors in addition to patients’ pulmonary status. Diaphragmatic paralysis, the component with the most direct plausible surgical mechanism, was rare and occurred in 3/172 patients in the pulmonary artery vent group and in none of the 316 patients in the aortic root vent group; it therefore did not account for the lower respiratory composite rate observed with pulmonary artery venting. The respiratory composite finding should consequently be regarded as hypothesis-generating rather than as evidence of a definitive lung-protective effect. In addition, fast-track failure incorporates delayed extubation, the primary outcome, together with intensive care unit stay longer than one day, while reintubation contributes to both composite outcomes. These secondary outcomes are therefore correlated by construction and should not be interpreted as independent corroborating evidence.

### 4.4. Postoperative Atrial Fibrillation and Inotropic Support

Postoperative atrial fibrillation was less frequent in the pulmonary artery vent group in the unadjusted analysis, but this difference disappeared after overlap weighting. This contrasts with the findings of Gürer et al., who reported a lower incidence of postoperative atrial fibrillation with pulmonary artery venting and identified venting strategy as an independent factor in multivariable analysis [12]. The attenuation observed in our study suggests that the crude association was at least partly explained by substantial differences in baseline characteristics and operative profiles between groups. Because postoperative atrial fibrillation is multifactorial and is influenced by pre-existing patient characteristics, inflammation, oxidative stress, autonomic activation, atrial pressure, and perioperative management [19], the independent contribution of venting strategy is likely to be modest and requires confirmation in prospectively designed studies.

In contrast, postoperative inotrope requirement remained significantly lower in the pulmonary artery vent group after weighting. Effective decompression of the left heart during cardiopulmonary bypass could theoretically reduce ventricular distension and myocardial workload, thereby facilitating separation from bypass and early hemodynamic recovery [10]. Nevertheless, this interpretation remains speculative because direct intracardiac pressure measurements, myocardial injury biomarkers, ventricular function after bypass, inotrope dose and duration, and standardized criteria for initiating inotropic support were not available. The inotrope finding should therefore be regarded as an exploratory secondary observation rather than evidence of a direct myocardial-protective effect.

### 4.5. Clinical Implications

From a clinical perspective, the present findings do not support changing venting strategy solely to reduce delayed extubation, because the primary outcome was not statistically significant after adjustment. Several secondary outcomes—including fast-track failure, the respiratory composite outcome, inotrope requirement, and PaO_2_ at extubation—showed favorable associations with pulmonary artery venting; however, these outcomes were exploratory, some were correlated by construction, and they should not be interpreted as independent evidence of superior postoperative recovery. Not all postoperative outcomes favored pulmonary artery venting. Although intensive care unit stay was shorter in the unadjusted analysis, median hospital stay was slightly longer in the pulmonary artery vent group (7 [6–7] vs. 6 [5–7.2] days); this difference was not evident after overlap weighting (adjusted mean difference 0.22 days, 95% CI −0.34 to 0.75; *p* = 0.440). Stroke was uncommon but numerically more frequent in the pulmonary artery vent group (4/172 [2.3%] vs. 2/316 [0.6%]). Given the small number of events and the observational design, this numerical difference cannot be attributed to the venting strategy, but it further argues against interpreting the overall findings as evidence of uniformly superior postoperative recovery with pulmonary artery venting.

Nevertheless, treatment decisions should remain individualized and should account for surgical anatomy, myocardial protection strategy, de-airing requirements, surgeon experience, and institutional practice. The results should not be interpreted as demonstrating superiority of pulmonary artery venting, because the study was observational, the primary outcome was neutral, and several favorable findings were secondary and exploratory. Prospective studies using standardized extubation protocols and predefined pulmonary and hemodynamic endpoints are needed before firm clinical recommendations can be made.

### 4.6. Strengths and Limitations

This study has several strengths. It included a relatively large cohort of patients undergoing on-pump CABG and evaluated both respiratory and broader early postoperative outcomes. Propensity-score overlap weighting was used to address marked baseline differences between venting groups. As expected from the algebraic properties of overlap weighting based on a logistic propensity-score model, exact balance was achieved in the first moments of the covariates included in the propensity model; this should not be interpreted as evidence that higher-order covariate structure or unmeasured confounding was eliminated. The propensity-score model was refitted in each bootstrap sample, providing confidence intervals that incorporated uncertainty in treatment-assignment modeling. In addition, the robustness of the primary outcome was examined using alternative delayed-extubation thresholds.

Several limitations should also be acknowledged. First, the retrospective, single-center design precludes causal inference and remains susceptible to residual and unmeasured confounding despite overlap weighting. Venting strategy was not randomized but primarily reflected the established practice of two concurrently active surgical teams, each of which consistently used a distinct venting strategy. Both strategies were represented throughout the study period, and their relative distribution did not differ significantly across calendar years. Nevertheless, because surgical team and venting strategy were intrinsically linked, the independent effect of the venting route cannot be fully separated from other unmeasured team-specific differences in case mix or perioperative practice. Overlap weighting addressed measured baseline differences but cannot eliminate residual confounding from unmeasured factors. In addition, validated baseline surgical risk scores, such as EuroSCORE II or the Society of Thoracic Surgeons (STS)score, were not routinely available in the retrospective dataset and therefore could not be incorporated into the adjustment strategy. Second, perioperative anesthetic management, ventilator weaning, extubation decisions, and indications for inotropic or respiratory treatments were based on routine clinical practice and may not have been fully standardized. Third, the respiratory composite outcome included supportive interventions such as nasotracheal aspiration and inhaled bronchodilator treatment, which may partly reflect clinician practice patterns rather than uniformly adjudicated pulmonary complications.

Fourth, oxygenation was assessed using PaO_2_ at extubation, but inspired oxygen concentration and PaO_2_/FiO_2_ ratios were unavailable. Direct measurements of pulmonary congestion, respiratory mechanics, intracardiac pressures, myocardial injury, and ventricular function after cardiopulmonary bypass were also not recorded. Fifth, several clinically important outcomes, including reintubation, stroke, and mortality, were uncommon, limiting the precision and stability of adjusted effect estimates. Although missingness was low for most variables, the valve-disease variable was excluded because 87.3% of observations were unavailable. Overlap weighting estimates effects in patients with the greatest comparability between treatment groups. Although all 488 participants were retained, Kish effective sample sizes were 135.0 for the aortic root vent group and 97.1 for the pulmonary artery vent group; the adjusted estimates should therefore be interpreted primarily for this clinical overlap population. Finally, the primary outcome did not reach statistical significance, and significant secondary findings should be considered exploratory in view of multiple outcome comparisons.

## 5. Conclusions

In patients undergoing on-pump CABG, pulmonary artery venting was associated with lower adjusted odds of fast-track failure, the respiratory composite outcome, and postoperative inotrope requirement, together with slightly higher PaO_2_ at extubation. However, delayed extubation beyond 8 h, the primary outcome, was not significantly different between venting strategies after overlap weighting. These findings suggest a possible association between pulmonary artery venting and more favorable early postoperative recovery, but they do not establish clinical superiority. Prospective multicenter studies with standardized perioperative protocols and predefined respiratory and hemodynamic outcomes are required to confirm these observations.

## Figures and Tables

**Table 1:** Baseline and procedural characteristics before and after overlap weighting.

Variable	Aortic Root Vent (*n* = 316)	Pulmonary Artery Vent (*n* = 172)	Absolute SMD Before Weighting	Aortic Root Vent Overlap-Weighted	Pulmonary Artery Vent Overlap-Weighted	Absolute SMD After Weighting
Age, years	60.73 ± 10.09	61.34 ± 10.04	0.060	61.48 ± 9.03	61.48 ± 10.35	0.000
Male sex	230 (72.8%)	97 (56.4%)	0.348	67.7%	67.7%	0.000
COPD	80 (25.3%)	53 (30.8%)	0.123	27.0%	27.0%	0.000
Diabetes mellitus	104 (32.9%)	117 (68.0%)	0.750	49.4%	49.4%	0.000
Hypertension	204 (64.6%)	52 (30.2%)	0.732	56.3%	56.3%	0.000
Hyperlipidemia	204 (64.6%)	137 (79.7%)	0.341	71.6%	71.6%	0.000
Previous myocardial infarction	38 (12.0%)	51 (29.7%)	0.445	22.4%	22.4%	0.000
Smoking history	180 (57.0%)	112 (65.1%)	0.168	61.9%	61.9%	0.000
Beta-blocker use	146 (46.2%)	74 (43.0%)	0.064	41.7%	41.7%	0.000
Calcium-channel blocker use	116 (36.7%)	36 (20.9%)	0.354	18.5%	18.5%	0.000
ACE inhibitor use	113 (35.8%)	17 (9.9%)	0.648	16.7%	16.7%	0.000
Aspirin use	117 (37.0%)	86 (50.0%)	0.264	36.9%	36.9%	0.000
Clopidogrel use	43 (13.6%)	6 (3.5%)	0.368	2.7%	2.7%	0.000
Preoperative BUN, mg/dL	25.62 ± 26.88	20.33 ± 8.03	0.267	20.37 ± 10.72	20.37 ± 7.73	0.000
Preoperative creatinine, mg/dL	1.07 ± 0.40	1.10 ± 0.25	0.113	1.10 ± 0.33	1.10 ± 0.24	0.000
Preoperative hematocrit, %	37.52 ± 7.28	40.82 ± 4.69	0.538	40.54 ± 5.78	40.54 ± 4.46	0.000
Left ventricular ejection fraction, %	51.03 ± 7.99	51.95 ± 8.56	0.110	51.56 ± 7.83	51.56 ± 8.24	0.000
NYHA functional class	2.03 ± 0.19	2.06 ± 0.26	0.144	2.04 ± 0.25	2.04 ± 0.28	0.000
Number of distal anastomoses	3.00 ± 0.91	2.42 ± 0.78	0.677	2.66 ± 0.91	2.66 ± 0.72	0.000
Emergency operation	24 (7.6%)	4 (2.3%)	0.244	4.6%	4.6%	0.000
Left main coronary artery disease	6 (1.9%)	4 (2.3%)	0.030	1.2%	1.2%	0.000
LIMA use	279 (88.3%)	162 (94.2%)	0.210	92.7%	92.7%	0.000

Data are presented as mean ± standard deviation or *n* (%). Overlap-weighted values represent the propensity-score-weighted pseudo-population. An absolute SMD < 0.10 was considered indicative of adequate covariate balance. Abbreviations: ACE, angiotensin-converting enzyme; BUN, blood urea nitrogen; COPD, chronic obstructive pulmonary disease; LIMA, left internal mammary artery; NYHA, New York Heart Association; SMD, standardized mean difference.

**Table 2:** Unadjusted intraoperative characteristics and postoperative outcomes.

Variable	Aortic Root Vent (*n* = 316)	Pulmonary Artery Vent (*n* = 172)	*p* Value
Intraoperative characteristics			
Aortic cross-clamp time, min	44.0 [35.0–61.2]	60.0 [45.0–71.5]	<0.001
Cardiopulmonary bypass time, min	90.0 [72.0–114.0]	85.0 [69.5–99.0]	0.019
Postoperative drainage, mL	440.0 [250.0–610.0]	500.0 [300.0–612.5]	0.142
Postoperative blood transfusion, units	2.0 [1.0–2.0]	1.0 [0.0–2.0]	<0.001
Intraoperative fluid balance, mL	100.0 [–300.0–662.5]	525.0 [150.0–800.0]	<0.001
Postoperative outcomes			
PaO_2_ at extubation, mmHg	98.2 [80.6–116.7]	105.0 [90.5–121.2]	<0.001
SaO_2_ at extubation, %	92.8 [90.0–97.0]	98.0 [95.0–99.0]	<0.001
Extubation time, h	9.0 [6.0–11.0]	7.5 [6.0–10.0]	0.001
Delayed extubation (>8 h)	169/314 (53.8%)	64/172 (37.2%)	<0.001
Intensive care unit stay, days	1.0 [1.0–2.0]	1.0 [1.0–1.0]	<0.001
Hospital stay, days	6.0 [5.0–7.2]	7.0 [6.0–7.0]	0.004
Fast-track failure	190/314 (60.5%)	70/172 (40.7%)	<0.001
Reoperation for bleeding	6/316 (1.9%)	5/172 (2.9%)	0.529
Postoperative atrial fibrillation	62/316 (19.6%)	17/172 (9.9%)	0.005
Respiratory composite outcome	137/316 (43.4%)	19/172 (11.0%)	<0.001
Inotrope requirement	76/316 (24.1%)	14/172 (8.1%)	<0.001
Low cardiac output syndrome	14/316 (4.4%)	6/172 (3.5%)	0.616
Acidosis	23/316 (7.3%)	6/172 (3.5%)	0.091
Nasotracheal aspiration	97/316 (30.7%)	6/172 (3.5%)	<0.001
Inhaled bronchodilator requirement	118/316 (37.3%)	16/172 (9.3%)	<0.001
Reintubation	16/316 (5.1%)	0/172 (0.0%)	0.003
Stroke	2/316 (0.6%)	4/172 (2.3%)	0.191
Mortality	16/316 (5.1%)	1/172 (0.6%)	0.010

Data are presented as median [interquartile range] or *n*/N (%). Continuous variables were compared using the Mann–Whitney U test; categorical variables were compared using Pearson’s chi-square test or Fisher’s exact test, as appropriate. Denominators vary because of missing data: aortic cross-clamp and cardiopulmonary bypass times were available for 316 and 314 aortic-root-vent patients and for 163 pulmonary-artery-vent patients; extubation time was available for 314 and 172 patients, respectively. Fast-track failure was defined as delayed extubation (>8 h), intensive care unit stay > 1 day, or reintubation. The respiratory composite outcome comprised diaphragmatic paralysis, nasotracheal aspiration, inhaled bronchodilator requirement, or reintubation. Abbreviations: PaO_2_, arterial oxygen partial pressure; SaO_2_, arterial oxygen saturation.

**Table 3:** Clinical outcomes after propensity-score overlap weighting.

Outcome	Aortic Root Vent Adjusted Estimate	Pulmonary Artery Vent Adjusted Estimate	Effect Estimate (Pulmonary vs. Aortic)	95% CI	*p* Value
Continuous outcomes					
Extubation time, h	9.00	8.50	MD −0.49	−1.48 to 0.41	0.269
Intensive care unit stay, days	1.39	1.20	MD −0.19	−0.39 to 0.03	0.085
Hospital stay, days	6.59	6.81	MD 0.22	−0.34 to 0.75	0.440
PaO_2_ at extubation, mmHg	98.77	105.85	MD 7.08	0.72 to 12.89	0.027
Binary outcomes					
Delayed extubation (>8 h)	51.4%	41.1%	OR 0.66	0.37 to 1.10	0.110
Fast-track failure	58.0%	42.2%	OR 0.53	0.30 to 0.89	0.016
Postoperative atrial fibrillation	13.4%	11.4%	OR 0.83	0.32 to 1.85	0.638
Respiratory composite outcome	35.0%	15.9%	OR 0.35	0.17 to 0.64	<0.001
Inotrope requirement	13.9%	6.0%	OR 0.39	0.14 to 0.87	0.027

Adjusted estimates were obtained using propensity-score overlap weighting. The propensity model included age, sex, chronic obstructive pulmonary disease, diabetes mellitus, hypertension, hyperlipidemia, previous myocardial infarction, smoking history, preoperative medications, blood urea nitrogen, creatinine, hematocrit, ejection fraction, NYHA functional class, number of distal anastomoses, emergency surgery, left main coronary artery disease, and left internal mammary artery use. Calendar year and surgical team were not included in the propensity-score model. Calendar-year distribution was examined separately and showed no significant difference in the relative use of the two venting strategies across the study period (Table S5), whereas surgical team was intrinsically linked to venting strategy. For continuous outcomes, the effect estimate is the adjusted mean difference; for binary outcomes, it is the adjusted odds ratio. Confidence intervals and *p* values were derived from 5000 stratified nonparametric bootstrap samples in which the propensity-score model was refitted and overlap weights were recalculated in each bootstrap sample. Delayed extubation was defined as extubation more than 8 h after surgery. Fast-track failure was defined as delayed extubation, intensive care unit stay longer than 1 day, or reintubation. The respiratory composite outcome comprised diaphragmatic paralysis, nasotracheal aspiration, inhaled bronchodilator requirement, or reintubation. Abbreviations: CI, confidence interval; MD, mean difference; OR, odds ratio; PaO_2_, arterial oxygen partial pressure.

## Data Availability

The anonymized data supporting the findings of this study are available from the corresponding author upon reasonable request. The analysis code used to reproduce the reported statistical analyses is provided as Supplementary File S1.

## Supplementary Materials

The following supporting information can be downloaded at: https://www.mdpi.com/article/10.3390/jcm15176655/s1, Table S1, Definitions and coding of study variables; Table S2, Summary and handling of missing data; Table S3, Propensity-score model and overlap-weighting diagnostics; Table S4, Sensitivity analyses using alternative thresholds for delayed extubation; Table S5, Distribution of venting strategies by calendar year; Table S6, Overlap-weighted estimates for prespecified secondary outcomes and components of composite outcomes; Figure S1, Study flow diagram; Figure S2, Propensity-score distribution by venting strategy; Figure S3, Distribution of extubation time by venting strategy; Supplementary File S1: Python analysis code and accompanying README file for the propensity-score overlap-weighted analyses, bootstrap inference, and sensitivity analyses.
