# Supplementary material for: Pulmonary Artery Versus Aortic Root Venting and Early Postoperative Respiratory Recovery After On-Pump Coronary Artery Bypass Grafting: A Propensity Score Overlap-Weighted Analysis
Source: J Clin Med. 2026 Aug 28;15(17):6655. doi: 10.3390/jcm15176655 (PMC13566017; doi:10.3390/jcm15176655)
Supplement: Supplementary file 1 [file jcm-15-06655-s001.zip › Table S3.pdf]

Supplementary Table S3. Propensity-score model and overlap-weighting diagnostics

Panel A. Multivariable logistic propensity-score model for pulmonary artery versus aortic root vent assignment

Covariate	Contrast	Adjusted OR	95% CI	P value
Age	Per 1-SD increase	1.29	0.96 to 1.75	0.095
Preoperative blood urea nitrogen	Per 1-SD increase	0.73	0.34 to 1.55	0.409
Preoperative creatinine	Per 1-SD increase	1.15	0.78 to 1.70	0.482
Preoperative hematocrit	Per 1-SD increase	1.90	1.30 to 2.77	<0.001
Left ventricular ejection fraction	Per 1-SD increase	1.29	0.95 to 1.75	0.102
NYHA functional class	Per 1-SD increase	1.35	1.03 to 1.76	0.028
Number of distal anastomoses	Per 1-SD increase	0.58	0.43 to 0.78	<0.001
Male sex	Yes vs no	0.43	0.23 to 0.78	0.005
Chronic obstructive pulmonary disease	Yes vs no	1.11	0.58 to 2.11	0.750
Diabetes mellitus	Yes vs no	5.28	2.97 to 9.40	<0.001
Hypertension	Yes vs no	0.13	0.07 to 0.23	<0.001
Hyperlipidemia	Yes vs no	2.35	1.29 to 4.27	0.005
Previous myocardial infarction	Yes vs no	4.80	2.39 to 9.62	<0.001
Smoking	Yes vs no	0.99	0.55 to 1.80	0.978
Beta-blocker use	Yes vs no	0.74	0.43 to 1.28	0.283
Calcium-channel blocker use	Yes vs no	0.67	0.34 to 1.32	0.248
ACE inhibitor use	Yes vs no	0.24	0.11 to 0.50	<0.001
Aspirin use	Yes vs no	3.70	2.06 to 6.64	<0.001
Clopidogrel use	Yes vs no	0.31	0.06 to 1.57	0.159
Emergency surgery	Yes vs no	0.48	0.12 to 2.04	0.323
Left main coronary artery disease	Yes vs no	1.74	0.19 to 16.35	0.626
Left internal mammary artery use	Yes vs no	1.15	0.40 to 3.33	0.801

Adjusted ORs describe the odds of assignment to the pulmonary artery vent group and are used only to construct the propensity score; they should not be interpreted as outcome effects. Continuous covariates were standardized before model fitting.

Panel B. Propensity-score and covariate-balance diagnostics

Diagnostic	Value	Interpretation
Participants included in the propensity model	488	All analysis covariates were complete.
Aortic root vent group	316	Exposure reference group.
Pulmonary artery vent group	172	Modelled treatment group.
Model convergence	Yes	Maximum-likelihood logistic regression converged.
McFadden pseudo-R^2	0.435	Propensity-model fit statistic.
Likelihood-ratio test	χ^2=275.58; P<0.001	Overall model test.
Aortic-root-group propensity score, median [IQR]	0.095 [0.031–0.241]	Estimated probability of pulmonary vent assignment.
Pulmonary-artery-group propensity score, median [IQR]	0.753 [0.523–0.920]	Estimated probability of pulmonary vent assignment.
Aortic-root-group propensity score range	0.000007–0.990	No trimming was applied.
Pulmonary-artery-group propensity score range	0.005600–0.997	No trimming was applied.
Near-extreme propensity scores (<0.05 or >0.95), aortic root vent group	108/316 (34.2%)	Descriptive overlap diagnostic.
Near-extreme propensity scores (<0.05 or >0.95), pulmonary artery vent group	35/172 (20.3%)	Descriptive overlap diagnostic.
Aortic-root-group overlap-weight range	0.000007–0.990	Weight = propensity score.
Pulmonary-artery-group overlap-weight range	0.002845–0.994	Weight = 1 − propensity score.
Effective sample size, aortic root group	135.0	Kish effective sample size after weighting.
Effective sample size, pulmonary artery group	97.1	Kish effective sample size after weighting.
Maximum absolute SMD before weighting	0.750	Largest baseline imbalance.
Covariates with absolute SMD >0.10 before weighting	19 of 22	Predefined balance threshold: 0.10.
Maximum absolute SMD after overlap weighting	<0.001	Numerical maximum was below 0.000001.
Covariates with absolute SMD >0.10 after weighting	0 of 22	All measured covariates met the balance criterion.
Participants retained after weighting	488	Overlap weighting downweighted rather than excluded observations.

Overlap weights were the propensity score for the aortic root vent group and 1 minus the propensity score for the pulmonary artery vent group. Near-extreme propensity scores were descriptively defined as <0.05 or >0.95. Absolute SMD <0.10 indicated adequate balance. ACE, angiotensin-converting enzyme; CI, confidence interval; IQR, interquartile range; NYHA, New York Heart Association; OR, odds ratio; SD, standard deviation; SMD, standardized mean difference.
