# Supplementary material for: Pulmonary Artery Versus Aortic Root Venting and Early Postoperative Respiratory Recovery After On-Pump Coronary Artery Bypass Grafting: A Propensity Score Overlap-Weighted Analysis
Source: J Clin Med. 2026 Aug 28;15(17):6655. doi: 10.3390/jcm15176655 (PMC13566017; doi:10.3390/jcm15176655)
Supplement: Supplementary file 1 [file jcm-15-06655-s001.zip › Table S4.pdf]

Supplementary Table S4. Sensitivity analyses using alternative thresholds for delayed extubation

Extubation threshold	Aortic vent adjusted risk	Pulmonary vent adjusted risk	Adjusted risk difference (95% CI)	Adjusted risk ratio (95% CI)	Adjusted odds ratio (95% CI)	P value
>6 h	70.5%	72.9%	2.4% (-9.2% to 13.2%)	1.03 (0.88 to 1.20)	1.13 (0.64 to 1.98)	0.667
>8 h (primary)	51.4%	41.1%	-10.3% (-23.9% to 2.4%)	0.80 (0.58 to 1.05)	0.66 (0.37 to 1.10)	0.110
>12 h	11.6%	8.1%	-3.6% (-11.6% to 4.2%)	0.69 (0.26 to 1.56)	0.67 (0.23 to 1.63)	0.339

Adjusted estimates were obtained using propensity-score overlap weighting. The >8-hour threshold was the prespecified primary definition of delayed extubation. Confidence intervals and P values were derived from 5000 stratified nonparametric bootstrap samples with refitting of the propensity-score model and recalculation of overlap weights in each sample. Effect estimates compare pulmonary with aortic venting; negative risk differences and values below 1.0 favor pulmonary venting. CI, confidence interval.
