# Supplementary material for: Pulmonary Artery Versus Aortic Root Venting and Early Postoperative Respiratory Recovery After On-Pump Coronary Artery Bypass Grafting: A Propensity Score Overlap-Weighted Analysis
Source: J Clin Med. 2026 Aug 28;15(17):6655. doi: 10.3390/jcm15176655 (PMC13566017; doi:10.3390/jcm15176655)
Supplement: Supplementary file 1 [file jcm-15-06655-s001.zip › Table S5.pdf]

Table S5. Distribution of venting strategies by calendar year.

Calendar year	Aortic root vent, n	Pulmonary artery vent, n	Total, n	Pulmonary artery vent, %
2020	38	22	60	36.7
2021	43	27	70	38.6
2022	68	37	105	35.2
2023	74	41	115	35.7
2024	84	41	125	32.8
January 2025	9	4	13	30.8
**Total**	**316**	**172**	**488**	**35.2**

Note: Both surgical teams were active concurrently throughout the study period. The study period extended through January 2025. Pearson's chi-square test showed no significant association between calendar year and venting strategy ($\chi^2 = 0.84$, df = 5, P = 0.974).
