# Supplementary material for: Pulmonary Artery Versus Aortic Root Venting and Early Postoperative Respiratory Recovery After On-Pump Coronary Artery Bypass Grafting: A Propensity Score Overlap-Weighted Analysis
Source: J Clin Med. 2026 Aug 28;15(17):6655. doi: 10.3390/jcm15176655 (PMC13566017; doi:10.3390/jcm15176655)
Supplement: Supplementary file 1 [file jcm-15-06655-s001.zip › Table S6.pdf]

Supplementary Table S6. Overlap-weighted estimates for additional prespecified secondary outcomes and components of composite outcomes

Outcome	Aortic root vent adjusted estimate	Pulmonary artery vent adjusted estimate	Effect estimate (pulmonary vs aortic)	95% CI	P value
Additional prespecified secondary outcomes					
SaO_2 at extubation, %	92.67	96.11	MD 3.44	2.38 to 4.41	<0.001
Postoperative transfusion, units	1.74	0.92	MD −0.83	−1.08 to −0.57	<0.001
Postoperative drainage, mL	542.07	525.42	MD −16.65	−99.12 to 67.90	0.679
Low cardiac output syndrome	3.7%	3.3%	OR 0.89	0.14 to 5.82	0.909
Acidosis	4.1%	3.3%	OR 0.80	0.12 to 3.73	0.761
Reoperation for bleeding	3.4%	1.9%	OR 0.55	0.07 to 12.55	0.491
Stroke	1.4%	2.4%	RD +1.04 pp	−2.35 to 4.80	0.505
Reintubation	4.9%	0.0%	RD −4.94 pp	−8.69 to −1.56	<0.001
In-hospital mortality	1.2%	1.1%	RD −0.08 pp	−2.26 to 2.24	0.741
Components of the respiratory composite outcome					
Diaphragmatic paralysis	0.0%	2.4%	RD +2.36 pp	0.00 to 5.99	0.098
Nasotracheal aspiration	18.3%	5.0%	OR 0.23	0.03 to 0.57	0.002
Inhaled bronchodilator requirement	30.2%	13.5%	OR 0.36	0.16 to 0.69	0.003
Components of fast-track failure					
Delayed extubation (>8 h)	51.4%	41.1%	OR 0.66	0.37 to 1.10	0.110
ICU stay >1 day	24.7%	8.6%	OR 0.29	0.12 to 0.56	<0.001

Adjusted estimates were obtained using the same 22-covariate propensity-score overlap-weighting model as in the primary analysis. Continuous outcomes are presented as overlap-weighted means and mean differences (MDs). Binary outcomes are presented as overlap-weighted risks; odds ratios (ORs) compare pulmonary artery with aortic root venting. For very sparse outcomes with fewer than five observed events in either group (stroke, reintubation, in-hospital mortality, and diaphragmatic paralysis), weighted risk differences (RDs) in percentage points (pp) are reported because bootstrap OR distributions were unstable or non-estimable; reintubation had no observed events in the pulmonary artery vent group, and diaphragmatic paralysis had no observed events in the aortic root vent group. Confidence intervals and P values were derived from 5000 stratified nonparametric bootstrap samples with refitting of the propensity-score model and recalculation of overlap weights in each sample. Reintubation is a component of both the respiratory composite outcome and fast-track failure and is reported in the first panel. Composite components were non-mutually exclusive. CI, confidence interval; ICU, intensive care unit.
