# Supplementary material for: Pulmonary Artery Versus Aortic Root Venting and Early Postoperative Respiratory Recovery After On-Pump Coronary Artery Bypass Grafting: A Propensity Score Overlap-Weighted Analysis
Source: J Clin Med. 2026 Aug 28;15(17):6655. doi: 10.3390/jcm15176655 (PMC13566017; doi:10.3390/jcm15176655)
Supplement: Supplementary file 1 [file jcm-15-06655-s001.zip › Table S1.pdf]

Supplementary Table S1. Definitions and coding of study variables

Variable	Definition	Coding or unit
Exposure		
Venting strategy	Cardiac venting technique used during on-pump coronary artery bypass grafting.	Aortic vent or pulmonary artery vent
Baseline characteristics		
Age	Age at the time of surgery.	Years
Sex	Biological sex recorded in the hospital file.	Male or female
Chronic obstructive pulmonary disease	Preoperative diagnosis of chronic obstructive pulmonary disease.	0 = No; 1 = Yes
Diabetes mellitus	Preoperative diagnosis of diabetes mellitus.	0 = No; 1 = Yes
Hypertension	Preoperative diagnosis of hypertension.	0 = No; 1 = Yes
Hyperlipidemia	Preoperative diagnosis of hyperlipidemia.	0 = No; 1 = Yes
Previous myocardial infarction	History of myocardial infarction before the index operation.	0 = No; 1 = Yes
Smoking	Current or previous smoking recorded in the medical file.	0 = No; 1 = Yes
Preoperative medications	Medication use before surgery, including beta-blockers, calcium-channel blockers, angiotensin-converting enzyme inhibitors, aspirin, and clopidogrel.	0 = No; 1 = Yes
Blood urea nitrogen	Most recent blood urea nitrogen value before surgery.	mg/dL
Creatinine	Most recent serum creatinine value before surgery.	mg/dL
Hematocrit	Most recent hematocrit value before surgery.	%
Ejection fraction	Preoperative left ventricular ejection fraction.	%
NYHA functional class	Preoperative New York Heart Association (NYHA) functional class recorded before surgery |	Ordinal class
Emergency surgery	Operation performed under emergency conditions.	0 = No; 1 = Yes
Left main coronary artery disease	Documented significant left main coronary artery disease.	0 = No; 1 = Yes
Left internal mammary artery use	Use of the left internal mammary artery as a graft.	0 = No; 1 = Yes
Intraoperative characteristics		
Number of anastomoses	Total number of coronary graft anastomoses performed.	Count
Aortic cross-clamp time	Duration of aortic cross-clamping.	Minutes
Cardiopulmonary bypass time	Total duration of cardiopulmonary bypass.	Minutes
Intraoperative fluid balance	Net intraoperative fluid balance.	mL
Postoperative outcomes		
Extubation time	Time from completion of surgery to tracheal extubation.	Hours
Delayed extubation	Tracheal extubation occurring more than 8 hours after surgery.	0 = No; 1 = Yes
Fast-track failure	Presence of at least one of the following: extubation after 8 hours, intensive care unit stay longer than 1 day, or reintubation.	0 = No; 1 = Yes
Intensive care unit stay	Duration of postoperative intensive care unit admission.	Days
Hospital stay	Duration of postoperative hospital admission.	Days
Postoperative atrial fibrillation	New atrial fibrillation documented after surgery.	0 = No; 1 = Yes
Respiratory composite outcome	Presence of at least one of the following: diaphragmatic paralysis, nasotracheal aspiration, inhaled bronchodilator requirement, or reintubation.	0 = No; 1 = Yes
Nasotracheal aspiration	Requirement for nasotracheal aspiration during the postoperative period.	0 = No; 1 = Yes
Inhaled bronchodilator requirement	Requirement for inhaled bronchodilator treatment during the postoperative period.	0 = No; 1 = Yes
Reintubation	Requirement for repeat tracheal intubation after initial extubation.	0 = No; 1 = Yes
Inotrope requirement	Requirement for postoperative inotropic support.	0 = No; 1 = Yes
Low cardiac output	Postoperative low cardiac output documented in the clinical record.	0 = No; 1 = Yes

Variable	Definition	Coding or unit
Acidosis	Postoperative acidosis documented in the clinical record.	0 = No; 1 = Yes
Revision	Return to the operating room for postoperative surgical revision.	0 = No; 1 = Yes
Postoperative transfusion	Number of units of blood products administered postoperatively.	Units
Drainage	Total postoperative mediastinal/chest drainage.	mL
PaO2 at extubation	Arterial oxygen partial pressure measured at or immediately before extubation.	mmHg
SaO2 at extubation	Arterial oxygen saturation measured at or immediately before extubation.	%
Stroke	New postoperative stroke documented in the clinical record.	0 = No; 1 = Yes
Mortality	In-hospital postoperative death.	0 = No; 1 = Yes

ACE, angiotensin-converting enzyme; LIMA, left internal mammary artery; PaO2, arterial oxygen partial pressure; SaO2, arterial oxygen saturation.
