# Supplementary material for: Pulmonary Artery Versus Aortic Root Venting and Early Postoperative Respiratory Recovery After On-Pump Coronary Artery Bypass Grafting: A Propensity Score Overlap-Weighted Analysis
Source: J Clin Med. 2026 Aug 28;15(17):6655. doi: 10.3390/jcm15176655 (PMC13566017; doi:10.3390/jcm15176655)
Supplement: Supplementary file 1 [file jcm-15-06655-s001.zip › Table S2.pdf]

Supplementary Table S2. Summary and handling of missing data

Variable	Aortic vent (n=316)	Pulmonary vent (n=172)	Overall (n=488)	Handling in the statistical analysis
Aortic cross-clamp time	0 (0.0%)	9 (5.2%)	9 (1.8%)	Available-case analysis for analyses involving cross-clamp time.
Cardiopulmonary bypass time	2 (0.6%)	9 (5.2%)	11 (2.3%)	Available-case analysis for analyses involving cardiopulmonary bypass time.
Time to postoperative atrial fibrillation	22 (7.0%)	1 (0.6%)	23 (4.7%)	Used only for descriptive timing analyses; missing timing did not alter atrial fibrillation status.
Extubation time	2 (0.6%)	0 (0.0%)	2 (0.4%)	Patients with missing extubation time were excluded only from extubation-time analyses.
Delayed extubation (>8 h)	2 (0.6%)	0 (0.0%)	2 (0.4%)	Primary outcome was analysed in patients with available extubation time (n=486).
Fast-track failure	2 (0.6%)	0 (0.0%)	2 (0.4%)	Patients with missing extubation time were excluded from this composite outcome analysis.
Valve disease variable	276 (87.3%)	150 (87.2%)	426 (87.3%)	Not included in propensity-score or outcome models because of extensive missingness.

Values are missing n (%). All covariates included in the propensity-score model were complete; therefore, no multiple imputation was required for the primary overlap-weighted analysis. Outcome-specific available-case analysis was used.
